# Barth Syndrome: From Mitochondrial Dysfunctions Associated with Aberrant Production of Reactive Oxygen Species to Pluripotent Stem Cell Studies

**DOI:** 10.3389/fgene.2015.00359

**Published:** 2016-01-20

**Authors:** Ana Saric, Karine Andreau, Anne-Sophie Armand, Ian M. Møller, Patrice X. Petit

**Affiliations:** ^1^INSERM U 1124 “Toxicologie, Pharmacologie et Signalisation Cellulaire” and “FR 3567” CNRS Chimie, Toxicologie, Signalisation Cellulaire et Cibles Thérapeutiques, Université Paris Descartes – Centre Universitaire des Saints-PèresParis, France; ^2^Division of Molecular Medicine, Ruđer Bošković InstituteZagreb, Croatia; ^3^Department of Molecular Biology and Genetics, Aarhus UniversitySlagelse, Denmark

**Keywords:** barth syndrome, tafazzin, cardiolipin, mitochondria, stem cells, cellular models, mitochondrially targeted antioxidant

## Abstract

Mutations in the gene encoding the enzyme tafazzin, *TAZ*, cause Barth syndrome (BTHS). Individuals with this X-linked multisystem disorder present cardiomyopathy (CM) (often dilated), skeletal muscle weakness, neutropenia, growth retardation, and 3-methylglutaconic aciduria. Biopsies of the heart, liver and skeletal muscle of patients have revealed mitochondrial malformations and dysfunctions. It is the purpose of this review to summarize recent results of studies on various animal or cell models of Barth syndrome, which have characterized biochemically the strong cellular defects associated with *TAZ* mutations. Tafazzin is a mitochondrial phospholipidlysophospholipid transacylase that shuttles acyl groups between phospholipids and regulates the remodeling of cardiolipin (CL), a unique inner mitochondrial membrane phospholipid dimer consisting of two phosphatidyl residues linked by a glycerol bridge. After their biosynthesis, the acyl chains of CLs may be modified in remodeling processes involving up to three different enzymes. Their characteristic acyl chain composition depends on the function of tafazzin, although the enzyme itself surprisingly lacks acyl specificity. CLs are crucial for correct mitochondrial structure and function. In addition to their function in the basic mitochondrial function of ATP production, CLs play essential roles in cardiac function, apoptosis, autophagy, cell cycle regulation and Fe-S cluster biosynthesis. Recent developments in tafazzin research have provided strong insights into the link between mitochondrial dysfunction and the production of reactive oxygen species (ROS). An important tool has been the generation of BTHS-specific induced pluripotent stem cells (iPSCs) from BTHS patients. In a complementary approach, disease-specific mutations have been introduced into wild-type iPSC lines enabling direct comparison with isogenic controls. iPSC-derived cardiomyocytes were then characterized using biochemical and classical bioenergetic approaches. The cells are tested in a “heart-on-chip” assay to model the pathophysiology *in vitro*, to characterize the underlying mechanism of BTHS deriving from TAZ mutations, mitochondrial deficiencies and ROS production and leading to tissue defects, and to evaluate potential therapies with the use of mitochondrially targeted antioxidants.

## Introduction

Given the importance of cardiolipin (CL) for mitochondrial oxidative function and its role in many signaling pathways, it is not surprising that changes in CL biosynthesis are associated with a number of pathological conditions (Claypool and Koehler, 2012; Mejia et al., 2014a; Ren et al., 2014). Decreases in CL *per se* and decrease in CL content linked to changes in its acyl chain composition and/or CL peroxidation have been associated with a disruption of mitochondrial homeostasis in multiple tissues in diverse pathological conditions, including ischemia, hypothyroidism, aging (Shi et al., 2010), heart failure, ischemic reperfusion injury, and neurodegenerative disease (Chicco and Sparagna, 2007; Claypool and Koehler, 2012; Lu and Claypool, 2015). At the cellular level, CLs can promote membrane fusion processes critical for normal mitochondrial dynamics acting as a non-bilayer forming lipid (with calcium ions) and capable of affecting the function and activities of autophagic proteins (Chu et al., 2014). CLs have been implicated in mitophagy, the removal of damaged mitochondria, which is a critical step in mitochondrial quality control (Zhu et al., 2013). The accumulation of damaged mitochondria resulting from defective elimination by mitophagy is linked to deficient mitochondrial respiration and high levels of reactive oxygen species (ROS; Gandhi et al., 2009). CLs have also been implicated in apoptotic signaling and the crosstalk between mitochondria and the vacuole (Zischka et al., 2008). Finally, mitochondria can sense signals linked to changes in energy demand and respond to them by affecting nuclear gene expression possibly through changes in CL levels and acyl chain length (Jahnke et al., 2009).

In this review, we focus on the Barth Syndrome (BTHS), an X-linked disease which is characterized by CL deficiency and alteration of CL species as a result of mutation of the Tafazzin (*TAZ*) gene (Barth et al., 1981, 1983). We focus on recent progress in the field, dealing with the elucidation of tafazzin function and localization, CL synthesis and remodeling, multiple models of BTHS and recent developments achieved with induced pluripotent stem cells (iPSCs) from the tissues of patients with tafazzin gene mutations characteristically associated with BTHS, together with “heart-on-chip” technology.

Throughout this review we will cite studies on the model organism, baker’s yeast. The *Saccharomyces cerevisiae taz1* gene (*YPR140w*) is homologous to the human *TAZ* gene and highly conserved and the studies have had a considerable influence on our understanding of BTHS. Even more important has been the generation of human model systems: The differentiation of disease-relevant and patient-specific cell lines into relevant cell types have made it possible to generate unlimited amounts of appropriate cellular material for biochemical studies and mechanical measurements. However, for useful insight to be obtained with these tools, appropriate cell-based assays reproducing crucial aspects of the disease studied are required. For example, studies aiming to elucidate the link between aberrant ROS levels in mitochondria and defects in sarcomere organization and mechanical capacities have been driven by the development of cardiac muscle-derived human pluripotent stem cells and “heart-on-chip” technology.

## The Barth Syndrome

In a brief preliminary communication, Barth et al. (1981) described a family with a “new” X-linked syndrome affecting heart muscle, skeletal muscle and neutrophils. Barth et al. (1983), a large Dutch kindred was described with CM, skeletal myopathy and neutropenia, and with high infant mortality due to infection or cardiac failure. BTHS is considered to be a rare X-linked genetic disease characterized by CM, skeletal myopathy, growth retardation, neutropenia, and abnormally high levels of 3-methylglutaconic acid excretion in urine (Barth et al., 1983, 1999; Kelley et al., 1991; Spencer et al., 2006; Clarke et al., 2013) (**Table 1**). The authors also suggested that a similar disorder described by Neustein et al. (1979) as “an X-linked recessive CM with abnormal mitochondria” might be the same syndrome. Neustein et al. (1979) had reported that “A transvascular endomyocardial biopsy from an infant with CM and chronic congestive heart failure showed abnormal mitochondria when examined by electron microscopy. At necropsy, similar abnormal mitochondria were seen in skeletal muscles, liver, and kidney”. The ultrastructure of the affected tissues clearly suggested a mitochondrial disease (Barth et al., 1981).

**Table 1:** Clinical features of Barth syndrome (common features are indicated by an asterisk^∗^ and the most prominent ones are shown in red).

Cardiovascular	^∗^Dilated cardiomyopathy
	^∗^Left ventricular non-compaction


	Prolonged corrected QT interval


	Endocardial fibroelastosis


	Ventricular arrhythmia/Sudden cardiac death


	Undulating cardiomyopathy


	Hypertrophic cardiomyopathy (rarely)


Neuromuscular	^∗^Delayed motor milestones


	^∗^Proximal myopathy


	^∗^Abnormal fatigability


	^∗^Exercise intolerance


Neurological	^∗^Mild learning disabilities


	^∗^Attention deficits


	Strokes (cardiac embolic)


Hematological and Infectious	^∗^Neutropenia


	^∗^Recurrent mouth ulcers and sore gums


	^∗^Perianal dermatitis


	Recurrent bacterial infections


	Septicemia


Endocrine and Metabolic	^∗^3-Methylglutaconic aciduria


	^∗^Constitutional growth retardation with delayed bone age


	^∗^Delayed puberty


	Hypocholesterolemia


	Hypoglycemia


	Lactic acidosis (often accompanies cardiac failure)


	Osteopenia


Gastrointestinal	^∗^Oromotor feeding problems


	Episodic or chronic diarrhea


Dysmorphic features	^∗^Deep-set eyes


	^∗^Full cheeks


	^∗^Prominent ears (older boys)


Fetal	Cardiomyopathy


	Fetal hydrops


	Miscarriage and stillbirth of male fetuses




*Modified from Clarke et al. (2013).*

Mutations in the gene *TAZ*, encoding the phospholipid–lysophospholipid transacylase, cause BTHS (Bione et al., 1996; Barth et al., 2004). BTHS may be fatal if not diagnosed and treated early (Barth et al., 1983; Kelley et al., 1991; Johnston et al., 1997). 3-Methylglutaconic aciduria (MGA) is observed in a heterogeneous group of syndromes. It results from an increase in the excretion of 3-methylglutaconic and 3-methylglutaric acids, both of which are breakdown products of leucine catabolism (Wysocki and Hahnel, 1976).

**FIGURE 1:**
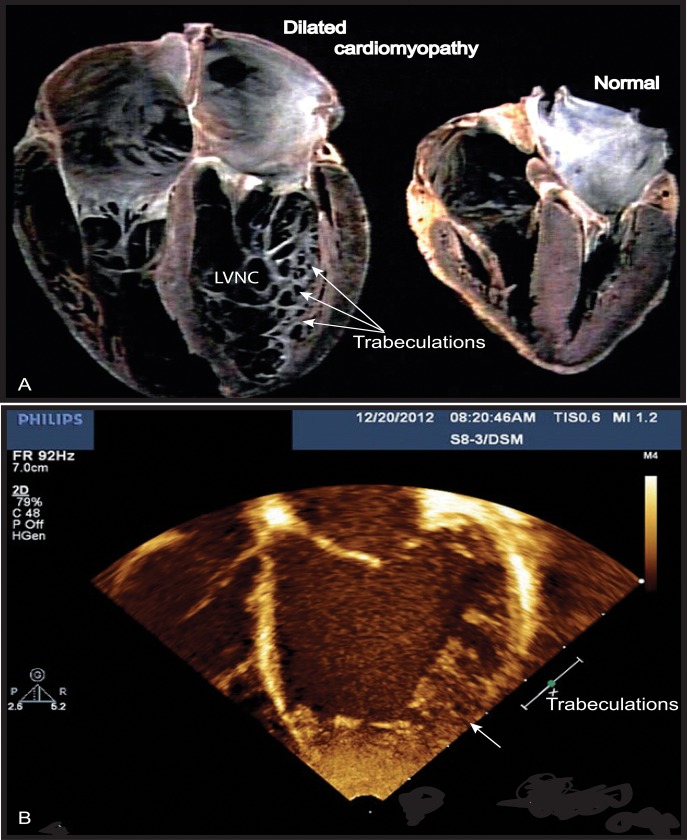
**Left ventricular non-compaction (LVNC) associated with a dilated cardiomyopathy phenotype in Barth syndrome (BTHS) patient.** **(A)** Example of a normal heart and a heart with dilated CM associated with LVNC. **(B)** BTHS patient with LVNC associated with dilated CM. Echocardiogram (apical 4-chamber view) of patient with BTHS depicting LVNC with associated DCM phenotype. The scale relates to Doppler effect velocity. Note the deep left ventricular trabeculations and dilated left ventricular chamber (Jefferies, 2013, with the permission of the editor, Wiley-Blackwell, John Wiley & Sons, USA).

BTHS generally presents as a CM affecting male individuals, often in infancy (**Figure 1**), although a case of BTHS was reported recently in a female patient as well (Cosson et al., 2012). Multiple additional abnormalities may be observed, including ventricular arrhythmia, sudden cardiac death, prolonged Q-Tc interval (the QT interval is an indicator of the electrical depolarization and repolarization of the ventricle), delayed motor milestones, proximal myopathy, lethargy, and fatigue, exercise intolerance, neutropenia (absent to severe; persistent, intermittent, or perfectly cyclic), compensatory monocytosis, recurrent bacterial infection, hypoglycemia, lactic acidosis, growth, and pubertal delay, feeding problems (poor appetite), failure to thrive, episodic diarrhea, characteristic facies, and an X-linked family history (Ichida et al., 2001; Steward et al., 2010).

**FIGURE 2:**
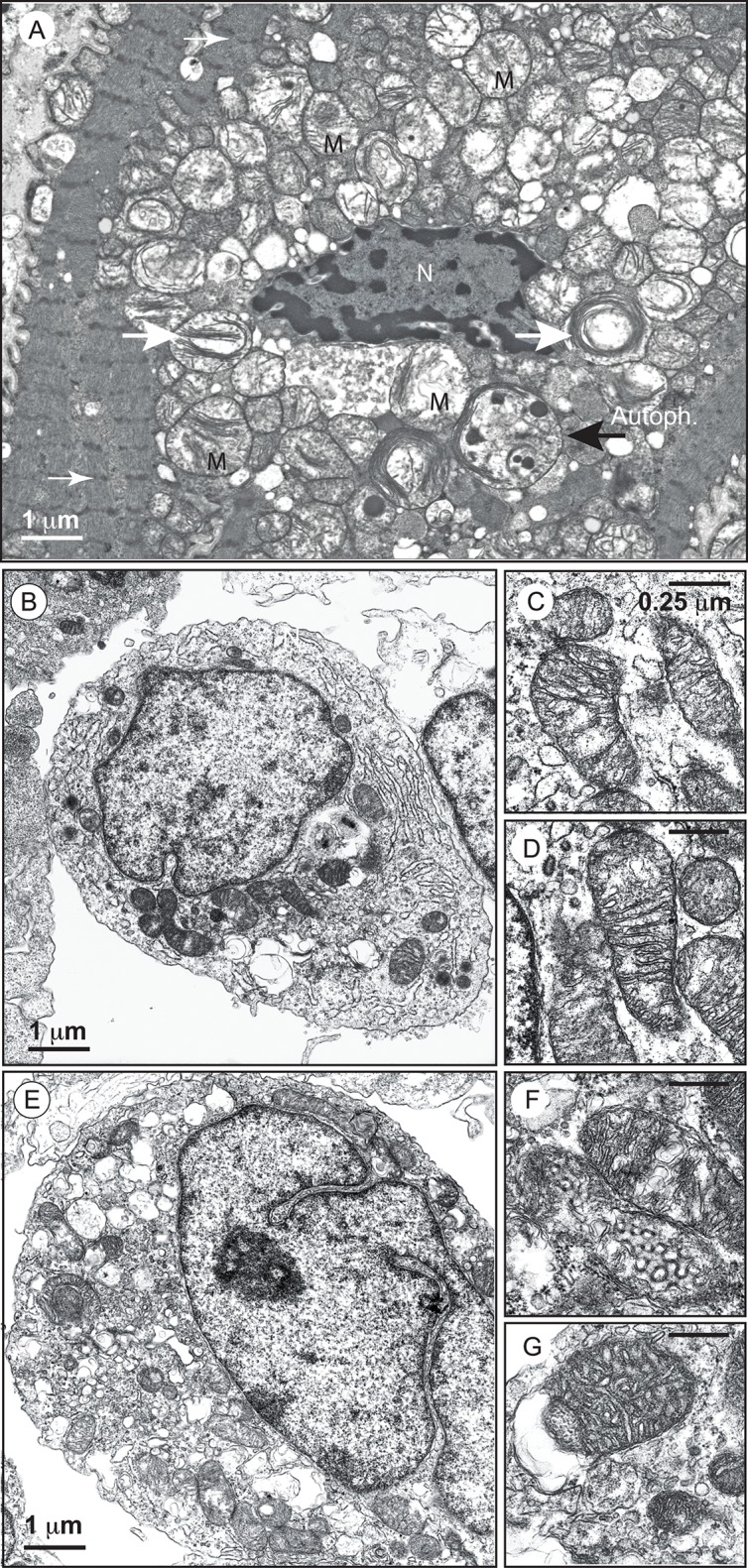
**Electron micrographs of mitochondria with an abnormal appearance in BTHS dilated cardiomyopathy and of lymphoblasts from a patient with Barth syndrome.** **(A)** Higher power electron micrograph. The mitochondria are enlarged and crowded together, with many touching each another. The cristae are not parallel, but are stacked, and many are organized into abnormal circular arrays (white big arrows). The nucleus (N) is centrally placed, but the myofilaments, with their cross-striations (small white arrows), are displaced to the periphery of the cell. Lamellar bodies are also dispersed within the cytoplasm. One autophagolysosome is also detectable (large black arrow; AutoPh.) [reprinted with the permission of Sarah Clarke et al. (2013) and BiomedCentral]. **(B)** Control lymphoblasts. **(C,D)** Structure of mitochondria from control lymphoblasts. **(E)** Lymphoblasts from a patient. **(F,G)** Structure of mitochondria from the lymphoblasts of a patient. Size bars are ^∗^Electron-translucent areas surrounded by the outer mitochondrial membrane (or a membrane derived from it). [from Dr. Patrice X. Petit, unpublished data and (Gonzalvez et al., 2013)].

Biopsies of the heart, liver, and skeletal muscles of patients reveal the presence of malformed mitochondria, with tightly stacked or circular bundles of cristae (Barth et al., 1983; Hodgson et al., 1987; Orstavik et al., 1998; Bissler et al., 2002). The mitochondria of lymphoblasts from patients have much less inner membrane than normal mitochondria, with collapsed cristae and a high frequency of fragmentation (Xu et al., 2005; Acehan et al., 2007; Gonzalvez et al., 2008, 2013). Similar abnormalities have been observed in HeLa-*TAZ* mutants (Gonzalvez et al., 2013) (**Figure 2**).

### Cardiomyopathy is a Major Feature of BTHS

Cardiomyopathy is the major clinical feature of BTHS, with a high incidence in early life. Prenatal losses or morbidity may also result from fetal CM (Steward et al., 2010). Patients may present dilated or hypertrophic CM, endomyocardial fibroelastosis, and/or a developmental CM known as myocardial non-compaction (left ventricular) or hypertrabeculation (“spongy myocardium”) (**Figure 1**) (Spencer et al., 2006; Clarke et al., 2013). It is usually of the dilated type and may be accompanied by endocardial fibroelastosis (Ades et al., 1993). During the development of dilated CM, cardiac myocytes undergo remodeling, including elongation, and death, whereas cardiac fibroblasts proliferate and their collagen secretion increases. Myocardial fibrosis has been reported in patients with BTHS (Neustein et al., 1979; Marziliano et al., 2007). About 50% of patients have prominent left ventricular trabeculations, suggestive of a form of left ventricular non-compaction (LVNC). Left ventricular abnormalities may be remodeled with age. There may also be a transition between relatively dilated and hypertrophic morphologies, sometimes referred to as an “undulating” phenotype, in children with LVNC (Pignatelli et al., 2003; Hanke et al., 2012). There is also a risk of ventricular arrhythmia and sudden death in BTHS, and this risk appears to be independent of CM severity (Ades et al., 1993; Barth et al., 2004; Yen et al., 2008). Spencer et al. (2006) found that 13% of patients had documented ventricular arrhythmia requiring defibrillator implantation (Roberts et al., 2012). BTHS patients may also suffer sudden cardiac death, as illustrated by the instances of sudden cardiac death in the family histories of two BTHS patients with documented arrhythmias (Spencer et al., 2005). Prolonged or borderline prolonged Q-Tc interval (the usual Q-Tc interval, the repolarization time between two heart beats, is ≤440 ms) is found in a high proportion of BTHS patients (43%), although this did not appear to be correlated with documented episodes of ventricular arrhythmia. A longer Q-Tc interval is associated with a higher risk of “torsade de pointes”, and borderline prolonged long Q-Tc intervals have been found in children with hypertrophic CM and dilated CM due to other causes, potentially reflecting the underlying cardiac muscle abnormalities, which include myofibrillar disorganization in BTHS (Spencer et al., 2006). CM is a common clinical presentation of mitochondrial diseases, and mitochondrial dysfunction has been identified as the probable cause of the CM observed in BTHS (Merante et al., 1994; Bunse et al., 2003; Sproule and Kaufmann, 2008). Clarke and coworkers (Clarke et al., 2013) recommended systematic screening for BTHS in male neonates, babies and young boys with dilated cardiomyopathy (DCM) or left LVNC (**Figure 1**), and in males with unexplained ventricular arrhythmia or sudden death family history.

## Tafazzin Introduced Briefly

The causal gene of BTHS, *TAZ*, encodes tafazzin (*TAZ*, G4.5, OMIM 300394) and was first discovered as the genetic origin of Barth syndrome Bione et al. (1996). The group responsible for identifying the tafazzin gene named it after a comic masochistic character (“tafazzi”) from an Italian television sports show. The tafazzin gene/protein has certainly lived up to its name (Houtkooper et al., 2009).

Tafazzin is a ubiquitous mitochondrial enzyme responsible for specific remodeling of the CLs by catalyzing acyl transfer from a phospholipid to a lysophospholipid. Since changes in CL amount and composition is central to the effect of tafazzin, it is necessary to first take a detailed look at the biosynthesis of CL and its role in the mitochondria before we can discuss the effect of tafazzin function and tafazzin mutations on cellular biochemistry and physiology.

## Cardiolipin

Decades of successful studies on the structural roles of lipids in membranes have resulted in the elucidation of the pathways by which these compounds are biosynthesized and the complex networks by which they are regulated. Until very recently, mitochondrial phospholipids attracted less attention. However, the identification of mitochondria as organelles involved not only in bioenergetic functions, but also in multiple regulatory and coordinating tasks, has focused research efforts on phospholipid signaling within and outside mitochondria especially in connection with apoptosis, autophagy and cell cycle regulation. These new insights are also linked to a greater awareness of the importance of the physical properties of the membrane for membrane function, including fluidity, defects, lateral mobility, and curvature strain (Kagan et al., 2014).

Mitochondrial membranes have a lower phospholipid-to-protein ratio than other organelle membranes, a high phosphatidylcholine (PC) and phosphatidylethanolamine (PE) content (together accounting for 80% of the total phosphorus present), low levels of sterols and sphingolipids, and a strong enrichment of a single phospholipid, CL (Horvath and Daum, 2013). The major polyglycerophospholipid in mammalian tissues is bis-(1,2-diacyl-sn-glycero-3-phospho)-1′-3′-sn-glycerol, or CL. CL was first isolated from beef heart (Pangborn, 1942), and its biosynthesis in mammals was first described in rat liver (Hostetler et al., 1971). It is found predominantly in the inner, and, to a lesser extent, outer membranes of mitochondria in non-pathological situations (Ardail et al., 1990; Hovius et al., 1990, 1993), and it is particularly abundant in the so-called “mitochondrial contact sites (MCSs)” in which the outer and inner membranes come into contact (Nicolay et al., 1990). MCSs have diverse functions in the regulation of apoptosis, organelle dynamics, cellular trafficking, and the immune response (Nicolay et al., 1990).

**FIGURE 3:**
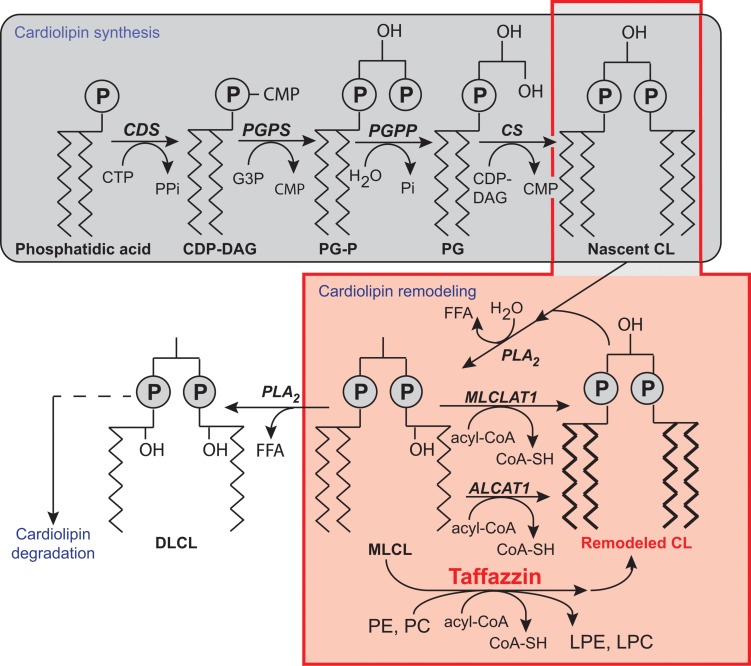
**Biosynthesis and remodeling of cardiolipin.** Putative pathways of CL biosynthesis, remodeling, and degradation in mammalian cells. Note that the length of the acyl chains in the final phospholipids does not reflect their true length, which is typically 16C, 18C, or 22C, nor their degree of unsaturation. Abbreviations: ALCAT 1, Acyl-CoA:lysocardiolipin acyltransferase 1; CDP-DAG, cytidine diphosphate-diacylglycerol; CDS, CDP-DAG synthase; CS, CL synthase; CMP, cytidine monophosphate; CTP, cytidine triphosphate; DLCL, dilyso-CL; FFA, free fatty acid; G3P, glycerol-3-phosphate; LPC, lysophosphatidyl choline; LPE, lysophosphatidylethanolamine; MLCL, monolyso-CL; MLCLAT 1, MLCL acyltransferase 1; PC, phosphatidylcholine; PE, phosphatidylethanolamine; PG, phosphatidylglycerol; PG-P phosphatidylglycerol phosphate; PGPP, phosphatidylglycerol phosphate phosphatase; PGPS, phosphatidylglycerol phosphate synthase; P_i_, inorganic phosphate; PLA_2_, phospholipase A_2_; tafazzin, transacylase. [Reprinted, with permission of the authors and modified from (Chicco and Sparagna, 2007; Mejia et al., 2014b; Ren et al., 2014) and with information from Claypool and Koehler (Claypool and Koehler, 2012), and also from the American Journal of Cell Physiology, edited by the American Physiology Society, USA).

Cardiolipin has several original properties, including a specific chemical structure, with four acyl chains displaying highly specific sensitivity to Ca^2+^, which can induce an inverted hexagonal lipid phase in the pure lipid. CLs consist of two phosphatidylglycerol units connected via a glycerol backbone, so as to form a dimeric structure (**Figure 3**). The polar “head” carries two negative charges, whereas the hydrophobic “tails” bear four acyl chains. The polar head has remained unmodified during the course of evolution, but the fatty acid residues have undergone modifications to both their chain length and degree of unsaturation. The eukaryotic CLs have long acyl chains and are polyunsaturated (18:2, 18:3, 22:6), whereas CLs from prokaryotes have much shorter acyl chain lengths and are fully saturated or mono-unsaturated. These differences reflect an adaptation to the transition from anaerobic to aerobic metabolism – the polyunsaturated fatty acid tails in eukaryotic CLs can undergo oxidation and be used in signaling (Tyurina et al., 2014a).

Most phospholipids are synthesized in the endoplasmic reticulum (ER), and some are then imported into the mitochondria [reviewed in (Hatch, 2004; Horvath and Daum, 2013)]. CL is unique because it is synthesized exclusively in the mitochondria [reviews: (Chicco and Sparagna, 2007; Ji et al., 2012; Kagan et al., 2014; Mejia et al., 2014b; Ren et al., 2014)]. In CL synthesis (**Figure 3**), once phosphatidic acid (PA) reaches the matrix side of the IMM, it is converted into cytidine diphosphate-DAG (CDP-DAG) and pyrophosphate, through reaction with cytidine triphosphate (CTP). This reaction is catalyzed by CDP-DAG synthase (CDS), the importance of which has been demonstrated in yeast ER and mitochondria (Kuchler et al., 1986; Shen et al., 1996). The conserved IMM-resident Tam41p is the mitochondrial CDP-DG synthase responsible for generating the CDP-DAG for CL biosynthesis (Tamura et al., 2006, 2013; Kutik et al., 2008; Tamura and Endo, 2013). CDS proteins in the ER provide the CDP-DAG for phospholipid biosynthesis, and TAMM41 supplies the CL pathway (Horvath and Daum, 2013). The downstream enzymes required for the biosynthesis of PG and CL are also located on the matrix-facing leaflet of the membrane. The commitment step in this pathway is catalyzed by phosphatidylglycerol phosphate (PGP) synthase (PGS1), which forms PGP from CDP-DAG and glycerol 3-phosphate (Chang et al., 1998a). PGP is then rapidly dephosphorylated to PG by PTPMT1 (Xiao et al., 2011; Zhang et al., 2011), an enzyme from the protein tyrosine phosphatase family displaying no primary sequence similarity to the yeast PGP phosphatase, Gep4p (Osman et al., 2010). In the steady state, PG is present at much lower levels (1–2%) than the other major mitochondrial phospholipids (Daum, 1985), consistent with rapid consumption of the newly synthesized PG by downstream pathways. Finally, cardiolipin synthase (CLS), an integral IMM protein with its active site facing the matrix (Schlame and Haldar, 1993), condenses PG with another molecule of CDP-DAG, to generate nascent unremodeled CL (Chang et al., 1998b; Chen et al., 2006; Lu et al., 2006). The defective acylation of CL with unsaturated fatty acids and a decrease in total CL levels are important biochemical signs of BTHS.

**FIGURE 4:**
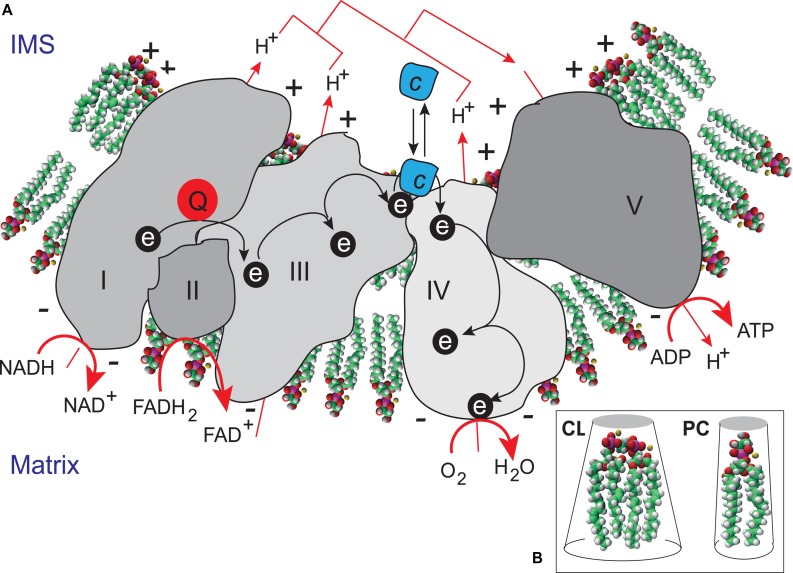
**Cardiolipin as a “glue” optimizing electron transport within the electron transport chain supercomplex.** **(A)** CL microlocalization on cristae membranes, “gluing” the respiratory complexes (Zhang et al., 2002) together to form supercomplexes, thereby decreasing the distance between redox partners. In addition to providing a platform for the aggregation of respiratory complexes, CL is also thought to serve as a proton trap (Haines and Dencher, 2002) on the outer leaflet of the IMM, mediating the rapid lateral diffusion of protons to the ATP synthase with minimal changes in bulk phase-pH. ADP, adenosine diphosphate; ATP, adenosine triphosphate; FADH_2_, reduced flavin adenine dinucleotide; FAD^+^, oxidized flavin adenine dinucleotide; NADH, reduced nicotinamide adenine dinucleotide; NAD^+^ oxidized nicotinamide adenine dinucleotide. **(B)** CL is a dimeric phospholipid with a small acidic head group and four acyl chains, resulting in a conical structure and a large surface area. As a result of its conical shape, CL exerts lateral pressure on a membrane containing other phospholipids, such as phosphatidylcholine (PC), resulting in curvature of the membrane and the proteins it contains.

**FIGURE 5:**
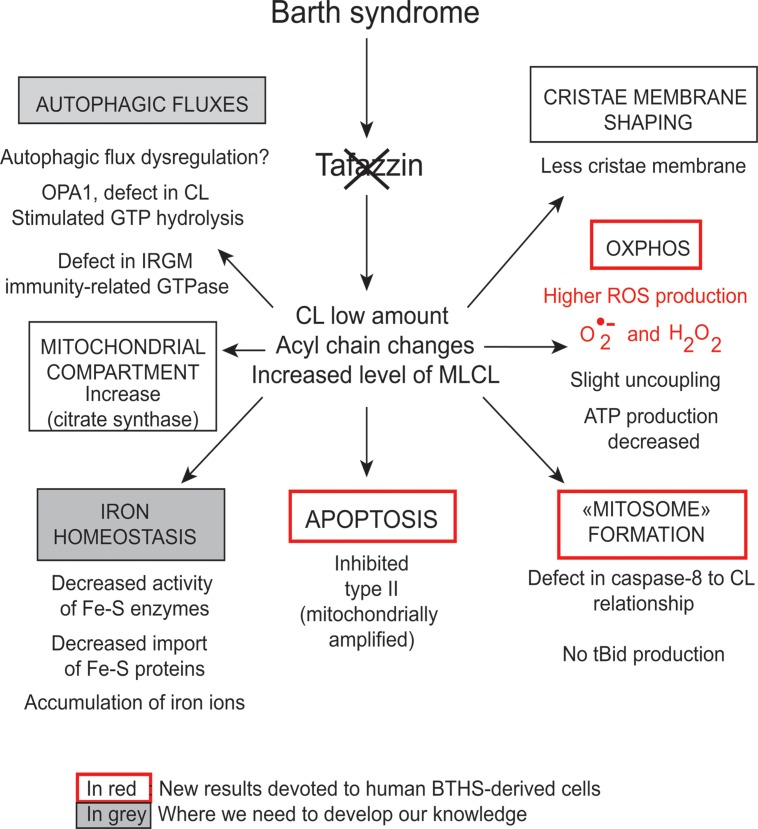
**Functions of cardiolipins, modifiers of Barth syndrome.** CL is involved in a plethora of cellular events and signaling pathways. The tafazzin mutations affect the CL acyl chain length and its degree of saturation and the synthesized CLs stay saturated instead of being converted into mature CL with unsaturated acyl chains. This impacts all CL-related functions that are listed in the Figure. IRGM; Immunity-related GTPase family, M [(human)], OPA1; Dynamin-like 120 kDa protein, mitochondrial encoded by the gene *OPA1*.

The functions of CLs initially identified were associated with their structural roles in the organization of lipid bilayers and definition of the curvature properties of membranes, together with their ability to interact with components of the respiratory complexes (**Figure 4**). CLs have recently been shown to associate with and modify the functions of several proteins, inn particular, a number of key IMM enzymes involved in the respiratory chain (RC), including cytochrome *c* oxidase and ATP synthase, carnitine palmitoyl-transferase, creatine phosphokinase, the pyruvate translocator, mono-, di-, and tricarboxylate carriers, glycerol-3-phosphate dehydrogenase, the phosphate transporter, and ATP/ADP translocase (Ardail et al., 1990; Schlame et al., 2000; Houtkooper and Vaz, 2008). A recent study used a molecular dynamics simulation model of the cytochrome *c* oxidase complex (complex IV) to show that there are precise CL binding sites at the entrance to the proton channels on the matrix side of the complex. Given the ability of CL to trap protons, the authors suggested that CL might also play an active role in transporting protons across complex IV to the intermembrane space (Arnarez et al., 2013). CL may be considered to function as a type of “glue”, holding the mitochondrial RC supercomplexes together to ensure that electron flow and proton transport are as efficient as possible (Zhang et al., 2002) (**Figure 4**). CLs are absolutely required for the activity of some of these respiratory enzymes, and their interactions with mitochondrial proteins are specific. The activity of these enzymes is not fully reconstituted if the CL is replaced with other phospholipids (Schlame and Ren, 2009; Ren et al., 2014). As CL may regulate ATP generation in cells, the maintenance of appropriate amounts of CL in the mitochondria is essential for correct mammalian cell function. This finding is consistent with the hypothesis that CL is responsible for maintaining the structural integrity of the proton-conducting protein environment and directly involved in proton uptake. The maintenance of appropriate levels of CL in mitochondria is therefore essential for correct cell function (**Figure 5**).

The key contribution of mitochondria to metabolic pathways and cell death or autophagic mechanisms requires sophisticated signaling systems (Li et al., 2015), where various lipid mediators, derived from polyunsaturated fatty acids, are essential. A range of diversified polyunsaturated molecular oxidation products is produced from CL by interaction with the intermembrane space hemoprotein, cytochrome *c*. Various oxygenated CL species undergo phospholipase A_2_-catalyzed hydrolysis to generate oxygenated fatty acids, including well known lipid mediators, such as oxygenated linoleic and arachidonic acids and monolysocardiolipins (MLCLs). These reactions constitute a new biosynthetic pathway for the production of lipid mediators and this pathway is activated *in vivo* after acute tissue injury (Tyurina et al., 2014a,b).

**FIGURE 6:**
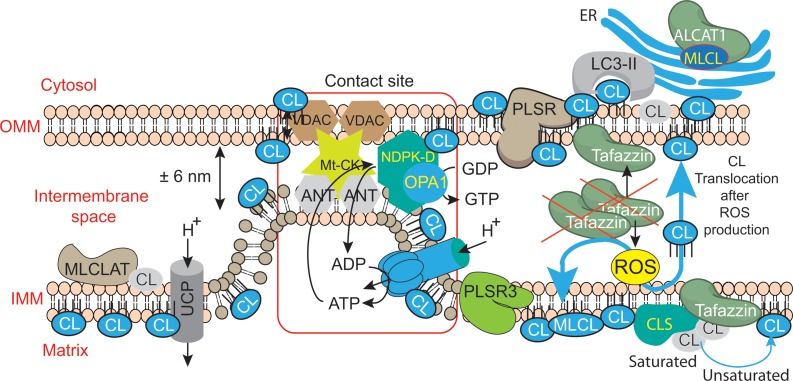
**Mitochondrial dysfunctions due to tafazzin mutations lead to ROS production, CL translocation and dysregulation of autophagic signaling.** CL is synthesized in the inner leaflet of the IMM by cardiolipin synthase (CLS). This is also the localization where it can be remodeled by tafazzin and monolysocardiolipin acyltransferase (MLCLAT), in a balanced system with PLA_2_. During processing or following stress signaling, CLs may also be translocated to the OMM and ER for remodeling, whereas in healthy mitochondria most of the CL remains at its site of synthesis in the IMM. The externalization of mature CL to the mitochondrial surface, through a mechanism involving phospholipid scramblase-3 (PLSR3), is a prerequisite for its recognition by light chain 3 protein (LC3) as a mitophagic “eat-me signal” targeting dysfunctional mitochondria to the autophagosomal machinery. The precise mechanism of scramblase regulation remains unclear, but its action may be physically facilitated by the proximity of nucleoside diphosphate kinase (NDPK-D). NDPK-D regulates the balance between di- and tri-phosphonucleotides, which provide GTP for the GTPase activity of a dynamin-like protein encoded by the gene OPA1 (for optic atrophy gene 1). By forming a hexamer in the intermembrane space, NDPK-D forms a physical bridge between the IMM and OMM. The switch from the phosphotransfer mode to a CL-translocating mode would thus facilitate the redistribution of CLs between the IMM and OMM. Note that immature CLs containing saturated acyl chains are gray while mature CLs with unsaturated acyl chains are blue. [Figure reproduced with modifications from Kagan et al. (2014), with the permission of the authors and of Elsevier Press].

However, CLs also play a key role in many other cellular processes (**Figure 4**), including apoptosis (Choi et al., 2007; Gonzalvez et al., 2008), autophagy (Chu et al., 2013, 2014), cell cycle regulation (He et al., 2013; Chao et al., 2014) and iron homeostasis (Patil et al., 2013). There are currently several lines of evidence to support the notion that mitochondria (with deleterious ROS production) are autophagic substrates capable of shaping autophagic responses in diverse ways (**Figure 6**) (DeVay et al., 2009; Ban et al., 2010; Rambold and Lippincott-Schwartz, 2011; Chu et al., 2013, 2014).

## Tafazzin Function

As already mentioned, tafazzin is a ubiquitous mitochondrial enzyme that catalyzes acyl transfer from a phospholipid to a lysophospholipid (**Figure 3**). The *TAZ* gene encodes for a protein or proteins responsible for specific remodeling of the CL to generate tetralinoleoyl cardiolipin (L_4_-CL), the principal constituent of CL in the heart.

Mammals have at least three enzymes capable of re-acylating MLCL: tafazzin [also cited by Xu et al. (2006) to be a “phospholipid-lysophospholipid transacylase”], MLCL acyltransferase 1 (MLCLAT1), and acyl-CoA:lysocardiolipin acyltransferase-1 (ALCAT1). Tafazzin is the only one of these three enzymes to have been conserved during evolution, from yeast to higher eukaryotes (Taylor and Hatch, 2003; Lu et al., 2006; Saini-Chohan et al., 2009; Claypool and Koehler, 2012). All the evidence obtained to date indicates that tafazzin is responsible for most of the CL remodeling occurring under physiological conditions. Tafazzin deficiency is characterized biochemically by an increase in MLCL (remodeling intermediate) levels, a decrease in CL (both the substrate and product) levels and an abnormal acyl chain profile in the remaining CL (Valianpour et al., 2005; Claypool et al., 2006, 2011; Houtkooper et al., 2009). However, the mechanism by which tafazzin establishes the steady-state acyl chain composition of CL remains unclear. tafazzin catalyzes a reversible transacylation reaction that displays no intrinsic acyl chain specificity, acts on acyl chains at both the *sn*-1 and *sn*-2 positions, and can use virtually any phospholipid and its lyso-derivatives as fatty acyl donor and acceptor, respectively (Lu et al., 2006; Malhotra et al., 2009).

Over 100 different pathogenic mutations have been identified throughout the gene giving rise to clinical signs. A list of known genetic variants is maintained by the Barth Syndrome Foundation https://www.barthsyndrome.org/science (Clarke et al., 2013). These mutations include missense and non-sense changes (Johnston et al., 1997), splicing defects, full or partial deletions and frame shifts. One of the main problems in studies of tafazzin is the alternative splicing of the primary transcript, resulting in different mRNAs and, potentially, different proteins (Bione et al., 1996). The tafazzin gene produces four main alternatively spliced mRNAs including full length (FL), exon 5 skipped (delta5, Δ5), exon 7 skipped (delta7,Δ7), and both exons 5 and 7 skipped (delta5/7, Δ5Δ7) (Kirwin et al., 2014). Kirwin et al. (2014). observed that a fraction of the minor splice variants can be predicted to be non-productive. Ronvelia et al. (2012) provided clear evidence that a single mutation can give rise to different clinical signs (phenotypes). Thus, although two patients had the same mutation, the infant displayed severe, characteristic features of BTHS (with congestive heart failure requiring heart transplantation at 11 months of age), whereas the great uncle presented only myopathy, not diagnosed until he was 43 years-old. This suggests that there are modifying factors that act as key determinants of the clinical progression of BTHS.

Expression studies with the tafazzin splice variants in tafazzin-deficient yeast (tazΔ) showed that only the human tafazzin variant lacking exon 5 could fully restore the aberrant CL profile of the tazΔ mutant. The full-length tafazzin variant only partially restored the CL profile. Protein sequence alignment suggested that exon 5 in the human tafazzin gene was introduced later in evolution, because it is absent from the corresponding genes of lower organisms (Vaz et al., 2003; Gonzalez, 2005). This hypothesis was confirmed by tafazzin gene analysis, which showed that the exon 5 sequence was present in all mammalian species tested, whereas the splice recognition site required for the incorporation of exon 5 into the mRNA was present only in primates. As CL composition is identical in primates (i.e., with mRNA transcripts containing exon 5) and other mammals (i.e., lacking exon 5), and the full-length human tafazzin was unable to restore the CL profile of tazΔ yeast fully, it is important to determine the function of this full-length variant in CL metabolism. BTHS patients with a mutation in exon 5 [“Human Tafazzin (TAZ) Gene Mutation and Variation Database”; www.barthsyndrome.org] have recently been identified. Theoretically, these patients should have normal levels of the tafazzin variant lacking exon 5 if the mutation does not render the initial transcript unstable or triggers its degradation. It would be very interesting to investigate the expression of the different tafazzin mRNA variants and more importantly the levels of CL and MLCL in these patients.

The presence of the various splicing variants and the apparent lack of correlation between the genotype and clinical phenotype of BTHS patients greatly complicate the task of designing experiments to understand BTHS pathogenesis (Johnston et al., 1997). Information about tafazzin mRNA levels, protein levels, and enzymatic activities, required for comparisons of genotype and phenotype, is missing. New findings are gradually emerging, but both quantitative protein analyses by immunoblotting and enzymatic activity measurements are still hampered by the experimental difficulties. A quantitative mRNA analysis has been developed for the different tafazzin splice variants, and this should make it possible to compare mRNA levels in the cells and/or tissues of control subjects and BTHS patients, and, therefore, determine whether there is at least a correlation between mRNA levels and genotype, if known. For instance, some tafazzin mutations may lead to an increase in transcription or the selective degradation of mRNAs.

## Tafazzin Localization and Organization

The localization of lipid biogenesis proteins within the mitochondrial membranes is not straightforward. CLS (*Crd1p*) was no exception, but was eventually localized to the matrix-facing leaflets of the inner membrane, where it catalyzes the synthesis of CL (Schlame and Haldar, 1993). We still know little about the submitochondrial location of endogenous mammalian tafazzin. Until very recently, the import into mitochondria and the assembly of tafazzin have only been studied in yeast. The yeast transacylase Taz1p was localized to the IMM and OMM facing the intermembrane space (Brandner et al., 2005; Claypool et al., 2006; Gebert et al., 2009; Xu et al., 2015). The Taz1p transacylase is imported into mitochondria and sorted to the OMM by a non-canonical targeting sequence, as it contains no typical N-terminal mitochondrial targeting sequence (Claypool et al., 2006). As mitochondrial targeting is undoubtedly driven by an internal targeting sequence, Taz1p probably inserts into a loop of the TOM complex facing the intermembrane space (Herndon et al., 2013). This situation appears to be radically different from that for the deacylase (*Cld1p*) reinitiating CL remodeling, which is located in the matrix leaflet of the IMM (Baile et al., 2013). This implies a more complex mechanism for the MLCL produced on the matrix side of the IMM in yeast. Due to its primary location, MLCL must either flip across the membrane or be transported to the OMM to gain access to tafazzin for remodeling (Baile et al., 2014). Tafazzin has been conserved as a CL remodeling enzyme in eukaryotes, suggesting that the mammalian tafazzin is likely to be located at a similar position within mitochondria. The localization of tafazzin might provide information about the complex mechanisms of CL and MLCL trafficking required to provide these molecules with access to tafazzin for remodeling (Baile et al., 2014). Tafazzin reacts indiscriminately with all phospholipid and lysophospholipid species, but it requires its substrates to be presented in a non-bilayer state (Schlame, 2013). This suggests that the propensity of CL to form non-bilayer structures is one of the key features underlying its suitability as the primary substrate of tafazzin. Tafazzin is anchored to the membrane by an internal peptide segment, which does not cross the membrane but protrudes into its hydrophobic interior (Claypool et al., 2006). Tafazzin appears to interact with protein complexes of various sizes (Claypool et al., 2006, 2008; Xu et al., 2015). Most of these complexes have an apparent mass of 400 kDa or less, but some tafazzin seems to be associated with larger supercomplexes that also contain the ADP-ATP-carrier and the ATP synthase (Claypool et al., 2008). Such supercomplexes form only if CL is present in the membrane. One recent study focused on tafazzin complexes extracted from isolated mitochondria from *Drosophila* and mammalian cell cultures (Xu et al., 2015). Tafazzin is thought to bind to multiple protein complexes in these organisms, in interactions that lack specificity. Very large tafazzin complexes were detected only in the presence of CL, whereas smaller complexes remained intact even after treatment with phospholipase A_2_. In mammalian cells, tafazzin has a half-life of only 3–6 h, much shorter than that of other mitochondrial proteins. This suggest that tafazzin is a transient resident of multiple protein complexes (Xu et al., 2015). Linked to that, it appears that during its short lifetime, tafazzin exchanges acyl groups between diverse membrane phospholipids, a reaction that is thought to support fluctuating membrane dynamics or cristae membrane assembly by lowering the energy of curvature formation (Schlame et al., 2012a). Effectively, tafazzin catalyzes an unusual type of reaction in terms of classical enzymology, as the signal transduction network is usually dominated by highly specific chemical reaction linked to and driven by a decrease in free energy. The enzymatic reaction driven by tafazzin is both non-specific and reversible. The main function of tafazzin appears to be conferring considerable “composition flexibility” on mixtures of lipids, through transacylation to generate molecules with the characteristics described above. The transacylation events minimize the energy of the lipid population more effectively than that of a particular species still in the lipid phase state. Indeed, tafazzin senses the conformation of the membrane through its curvature (controlled principally by CL insertion), selecting curvature-prone molecular species of CL (Gawrisch, 2012; Schlame et al., 2012a). Tafazzin may also interact with a protein complex formed by prohibitins and DNAJC19 for CL remodeling (Richter-Dennerlein et al., 2014).

A recent three-dimensional (3D) homology modeling of tafazzin allows the identification of amino acid residues correlated to CL binding and to mitochondrial membrane linkages that facilitate the acyl-transfer reactions (Hijikata et al., 2015). Exon 5 of the protein is predicted to be an intrinsically unstructured domain that may change the substrate affinity and/or determine the primate-specific molecular interactions. In contrast, exon 7 certainly encodes part of the “putative” substrate binding cleft because gene products lacking exon 7 lose the substrate binding capacity (Hijikata et al., 2015). It was also recently reported that tafazzin functional activity is required to provide mature CL that is selectively involved in the execution of mitophagic processes. Indeed, tafazzin deficiency caused defective mito-phagosome biogenesis, but did not affect other autophagic processes (Hsu et al., 2015). This is an important point that links straightforward tafazzin deficiency and CL alterations to impaired oxidative phosphorylation, ROS production and execution of specific autophagic events or, more specifically, mitophagic processes.

There is a major need for biochemical investigations to determine the submitochondrial localization of tafazzin at higher resolution and to answer key questions. For example, the precise site and context of CL remodeling remain to be determined. It remains unclear whether this process occurs at contact sites, at junctions between cristae, at the tips of cristae, or whether it is confined to hemifusion membrane zones. It would also be of interest to define the composition of the protein complexes with which tafazzin associates. Which proteins interact with tafazzin and what are their functions? CL remodeling must be considered in the context of CL flexibility and metabolism, and questions about the relationship between the sites of CL biosynthesis and remodeling remain to be answered. The timing of CL remodeling in terms of the life span of CL species may also be important. A role for biosynthetic enzymes other than tafazzin in remodeling cannot be ruled out. The lack of involvement of a phospholipase in CL remodeling (as tafazzin regenerates lysophospholipids, and monolysocardiolipin in particular) does not rule out the possible involvement of other enzymes, such as lipases and acyl transferases. Overall, the biological characteristics of tafazzin required for its function in CL remodeling remain unclear.

## CL Remodeling

As mentioned earlier, CL synthesis (**Figure 3**) in mammals occurs only in mitochondrial membranes and is evolutionarily conserved in Eukaryotes (Schlame and Haldar, 1993). Newly synthetized CL is produced in the inner leaflet of the IMM (**Figure 6**) (Schlame and Haldar, 1993; Dzugasová et al., 1998; Osman et al., 2010). The presence of CL in both leaflets of the IMM as well as in the OMM is now widely accepted and this final distribution of CL must involve trafficking steps even if the mechanisms and the players responsible for such processes are still poorly understood. Newly synthesized CL is immature, characterized by saturated acyl chains of variable length and asymmetry around the central carbon of the bridging glycerol (Schlame et al., 2005; Schlame and Ren, 2006). CL maturation therefore necessitates acyl chain remodeling. CL remodeling starts by the action of a phospholipase that removes an acyl chain forming MLCL, then reacylated by an acyl transferase or a transacylase to form mature CL (**Figure 3**). The mature Cl are characterized by their unsaturated acyl chains and a high degree of symmetry (Schlame et al., 2005; Claypool and Koehler, 2012).

Within the three distinct CL remodeling pathways described in mammals, tafazzin (Taz1p in yeast, the only remodeling enzyme identified there), is a MLCL transacylase which removes an acyl chain from another phospholipid (with a preference for PC or PE) and transfer it to MLCL, thus regenerating CL, but in a fully mature form (Xu et al., 2003, 2006). Two other enzymes are involved in mammalian CL remodeling. The MLCL acyltransferase 1 (MLCLAT1) in pig liver mitochondria exhibits a specificity for linoleate and resides on the inner leaflet of the IMM (**Figure 6**) (Taylor et al., 2012). Interestingly, the overexpression of MLCLAT1 in lymphoblasts derived from BTHS patients led to an increased incorporation of linoleic acid into CL. Conversely, RNAi knockdown of MLCLAT1 in Hela cells led to a lower incorporation of linoleic acid into CL (Taylor et al., 2012). However, a third enzyme, acyl-CoA:lysocardiolipin acyltransferase 1 (ALCAT1), is located in the Mitochondrial Associated Membrane of the ER (MAM) where phospholipid trafficking between the ER and the mitochondria takes place. ALCAT1 shows no specificity for linoleic acid and catalyzes the CL remodeling by incorporating long-chain polyunsaturated fatty acid chains into CL. The resulting CLs, more susceptible to oxidative damage, are associated with early CL peroxidation (Ng et al., 2005). Recently, ALCAT1 has been implicated in mitochondrial biogenesis (Li et al., 2012). Curiously, but not surprisingly considering its localization at the MAM complexes (OMM), overexpression of ALCAT1 causes oxidative stress, which leads to mitochondrial fragmentation and Mitofusin 2 depletion. Furthermore, ALCAT1 overexpression leads to mtDNA instability and depletion that are reminiscent of Mfn2 deficiency. Accordingly, the expression of Mfn2 rescues mitochondrial fusion and respiratory dysfunction (Li et al., 2012). ALCAT1 removal prevents mitochondrial fragmentation from oxidative stress by up-regulating Mfn2 expression, mtDNA copy number, and fidelity of mtDNA transcription (Liu et al., 2012). So, ALCAT1, which differs in localization from the two other CL remodeling enzymes, tafazzin and MLCLAT1, reveals an unexpected role of CL remodeling, linking oxidative stress to mitochondrial fusion defects.

## Barth Syndrome Models Bridging the Gap Between *TAZ* Mutations and the Physiopathological Features of the Disease

Several BTHS models are available, and they all display the characteristic biochemical defects underlying BTHS: high levels of MLCL, low levels of CL, and an abnormal acyl chain composition of the remaining CL, resulting in bioenergetic destabilization of the mitochondria.

### Pioneering Studies Using Yeast as a Model System

Genetic approaches to the investigation of BTHS mutations have greatly benefited from the yeast model (Joshi et al., 2009; Ye et al., 2014). Indeed, all the known missense mutations of the tafazzin gene were reconstituted in two sets of experiments. Claypool et al. (2011) established 21 different pathogenic missense mutations of *TAZ* in yeast Taz1p (*Saccharomyces cerevisiae*) (Claypool et al., 2011) out of which 18 could not replace functional endogenous tafazzin. Biochemical and cell characterization was subsequently carried out for the last three of the 21 yeast BTHS mutants, resulting in the establishment of three additional modes of tafazzin dysfunction (Whited et al., 2013) and the elucidation of their loss-of-function mechanisms. In summary, in-depth characterization of the yeast BTHS-mutant panel has identified seven functional classes of mutations found in BTHS, defined on the basis of their loss-of-function mechanisms (Claypool et al., 2006, 2011; Whited et al., 2013).

The seven classes of variants are: (1) non-functional truncated products resulting from frame shifts or aberrant splicing, (2) products that are mislocalized within mitochondria and prone to aggregation, (3) aberrantly assembled products, (4) catalytically dead products, (5) hypomorphic alleles encoding products with residual transacylase activity, (6) products unable to engage in stable productive assemblies, and (7) temperature-sensitive products.

The systematic screening of pathogenic variants has provided important mechanistic insights into the origin of the clinical heterogeneity of BTHS. It might be possible to translate the biochemical characteristics observed in yeast model systems into useful clinical applications for disease treatment if the defined loss-of-function mechanisms are conserved in an appropriate mammalian model.

Pioneering studies on yeast carried out in Greenberg’s laboratory and others (Chen et al., 2008) have suggested that there is a relationship between *TAZ* gene deletion, mitochondrial malfunction and ROS production, as indicated by the following evidence (**Table 2**):

**Table 2:** Consequences of *TAZ* mutations and subsequent tafazzin deficiency in multiple BTHS models.

Model organism	General phenotype	Mitochondrial events
Yeast *Saccharomyces cerevisiae*	• Temperature-sensitive growth on a non-fermentable carbon source	•Abnormal morphology
		• Partial uncoupling
		• Reduced osmotic stability
		•Increased oxidative stress ^∗^
		• Reduced stability of respiratory supercomplexes
		• Disruption of iron homeostasis
Fly *Drosophila melanogaster*	• Flight muscle weakness	•Abnormal morphology of the flight muscle
		• Defective spermatogenesis
		• Inner membrane aggregation
		• Reduced state-3 respiration
		• Reduced dimerization and dimer row formation by ATP synthase
Zebrafish	• Early lethality	
*Danio rerio*	• Abnormal embryogenesis and cardiac development	
Mouse	• Prenatal and perinatal mortality	• Hyperproliferation of cardiac mitochondria
*Mus musculus*	• Low body weight	•Abnormal morphology
	• Developmental cardiomyopathy	• Reduced crista density
	• Adult cardiomyopathy (dilated type)	• Disrupted alignment between mitochondria and myofibrils
	• Skeletal muscle weakness	
Human	• Fetal loss and stillbirth	•Abnormal morphology
*Homo sapiens*	• Abnormal growth	• Inner membrane aggregation
	• Chronic fatigue	• Smaller membrane potential (Δψ_m_)
	• Cardiomyopathy	• Reduced state-3 respiration
	• Skeletal muscle weakness	• Reduced stability of the respiratory RCs
	• Neutropenia	• Slight increase in ROS production
		• Release of cytochrome *c* and stimulation of apoptosis in progenitor cells
		• Resistance to Fas and TNFα-induced apoptosis
		• Disrupted alignment between mitochondria and myofibrils


*Very important clues from the yeast cell model are shown in red, whereas shared characteristics are shown in blue. ^∗^Indicates essential discoveries taken into account in the recent work on human induced pluripotent stem cells (hiPSC).*

- Subcellular fractionation showed that the taz1 protein was located exclusively in mitochondria in yeast (Ma et al., 2004).
- The yeast tafazzin protein displays a mitochondrial acyl-CoA dependent lysophosphatidylcholine (lyso-PC) acyltransferase activity related to triacylglycerol and mitochondrial lipid synthesis (Testet et al., 2005).
- Energy coupling in taz1Δ mitochondria is dependent on the rate of oxidative phosphorylation.
- Membrane stability is compromised in *taz1Δ* mitochondria exposed to high temperature and hypotonic conditions. This is also the case for the yeast CLS mutant, CRD1Δ (Zhong et al., 2004; Zhang et al., 2005). Coupling was measured in *taz1Δ* mutant cells containing different splice variants of the human *TAZ* gene. Only the variant that restores wild-type CL synthesis (lacking exon 5) restored coupling under hypotonic conditions and at high temperature (Ma et al., 2004).
- In response to ethanol, *taz1Δ* and *crd1Δ* mutants (Koshkin and Greenberg, 2000), which cannot synthesize CL, display high levels of protein carbonylation, an indicator of ROS production (Chen et al., 2008). The increase in ROS production was probably not due to defects in the defense systems protecting against oxidative stress, because the CL mutants were not sensitive to paraquat, menadione, or hydrogen peroxide (H_2_O_2_) (Chen et al., 2008). High levels of protein carbonylation can be rescued by oleic acid supplementation in the *taz1Δ* mutant, but not in the *crd1Δ* mutant. This suggests that oleoyl-CL and/or oleoyl-monolyso-CL allow the *taz1 Δ* mutant to grow in ethanol by decreasing oxidative stress. However, although RC dysfunction is usually considered to be a reason for searching for superoxide anions (Brand, 2010)and hydrogen peroxide production, interest in the correlation between tafazzin depletion and deleterious ROS production in BTHS is very recent (Gonzalvez et al., 2013).

Mitochondria are the site of iron-sulfur cluster biosynthesis, and the iron-sulfur clusters are exported to the rest of the cell to be used by a range of enzymes (Lill, 2009). Patil et al. (2013) showed that the loss of CL in the *crd1*Δ mutant led to an increase in the expression of iron uptake genes, associated with high levels of mitochondrial iron and high sensitivity to iron and hydrogen peroxide most likely due to the Fenton reaction producing the very reactive hydroxyl radical (Moller et al., 2011). Indeed, high mitochondrial iron levels result from the disruption of iron-sulfur (Fe-S) cluster biogenesis. Furthermore, *crd1*Δ cells have low levels of activity for mitochondrial Fe-S enzymes (aconitase, succinate dehydrogenase, and ubiquinol-cytochrome *c* oxidoreductase) and cytosolic Fe-S enzymes (sulfite reductase and isopropylmalate isomerase), demonstrating that the loss of CL affects mitochondrial Fe-S biogenesis. Although not entirely related, we can speculate that mitochondrial Fe-S biogenesis is disturbed in the *taz1Δ* mutant as well as in cells derived from human patients’ tissues.

In conclusion, research using the yeast cell model has provided a large body of results and hypotheses useful for future studies of BTHS, and greater use could be made of this system to investigate the pathogenesis of this rare disease.

### Barth Syndrome Models

Other whole-organism models including *taz*-depleted zebrafish (Khuchua et al., 2006), *taz^-^*^/^*^-^*fruit flies (Xu et al., 2006, 2009; Schlame et al., 2012b) and a mouse model of the BTHS are now available (Acehan et al., 2011; Soustek et al., 2011; Phoon et al., 2012). Many cellular models are available with patient-derived fibroblasts (Barth et al., 1996), lymphoblasts (Xu et al., 2005; Gonzalvez et al., 2008, 2013), neutrophils (Kuijpers et al., 2004; van Raam and Kuijpers, 2009), iPSCs (Dudek et al., 2013; Wang et al., 2014a) and *taz*-depleted rodents (Acehan et al., 2009; He, 2010; He et al., 2014). In both zebrafish (Khuchua et al., 2006) and *taz*-depleted mice (Acehan et al., 2011; Soustek et al., 2011; Phoon et al., 2012), the cardiac defects observed reproduce many of the relevant cardiac parameters noted in BTHS patients. In zebrafish, *TAZ* knockdown severely impairs development, and the degree of cardiac dysmorphology is proportional to the dose of morpholino administered (Khuchua et al., 2006). In addition to impaired cardiac function, *TAZ* knockdown mice have an abnormal skeletal muscle ultrastructure, with a disrupted sarcomeric spatial organization (Acehan et al., 2011). These muscle abnormalities are consistent with the data obtained for *taz^-/-^*fruit flies, which also have impaired muscle functions (Xu et al., 2006).

Many cellular models of BTHS display dysmorphic changes in mitochondrial morphology and energetic defects (Acehan et al., 2007, 2009; Gonzalvez et al., 2008, 2013), in patient-derived lymphoblasts (Xu et al., 2005; Gonzalvez et al., 2008, 2013), iPSCs (Dudek et al., 2013) and fibroblasts (Barth et al., 1996). These cell types also display low basal respiration levels, a low membrane potential, and compromised coupling of oxidative phosphorylation (OXPHOS). Respiratory supercomplexes (SCs) have abnormally low levels of enzymatic activity, and there is a shift in supercomplex assembly from large “respirasomes” to smaller, and presumably less efficient, SCs (McKenzie et al., 2006; Dudek et al., 2013; Gonzalvez et al., 2013). These alterations to RC assembly also increase ROS production.

However, each model has its own characteristics (**Table 2**). For example, the shRNA-inducible *TAZ* knockdown mouse displays a basal respiration that is lower than normal levels of maximal uncoupled respiration. Tafazzin knockdown mice display a marked impairment of oxygen consumption during exercise, with no significant effect on resting metabolic rates. CL deficiency results in a significantly lower than normal mitochondrial respiratory reserve capacity in neonatal cardiomyocytes. This decrease probably reflects the low level of activity of complex III, which requires CL for its assembly and optimal activity. These findings suggest a bioenergetic dysfunction similar to that observed in cell culture-based models. Changes in respiratory SC stability are suspected, but have yet to be demonstrated (Bowron et al., 2013).

The results from the various BTHS models indicate that the cardiolipin abnormalities that occur in the absence of tafazzin result in an OXPHOS dysfunction involving respiratory super-complex destabilization. These RC dysfunctions are associated with the production of ROS (mostly superoxide anions with local toxicity and, subsequently, hydroperoxides, which participate in deleterious signal transduction) (Gonzalvez et al., 2013). The “long-term” consequences of these effects may compromise heart development and function.

**FIGURE 7:**
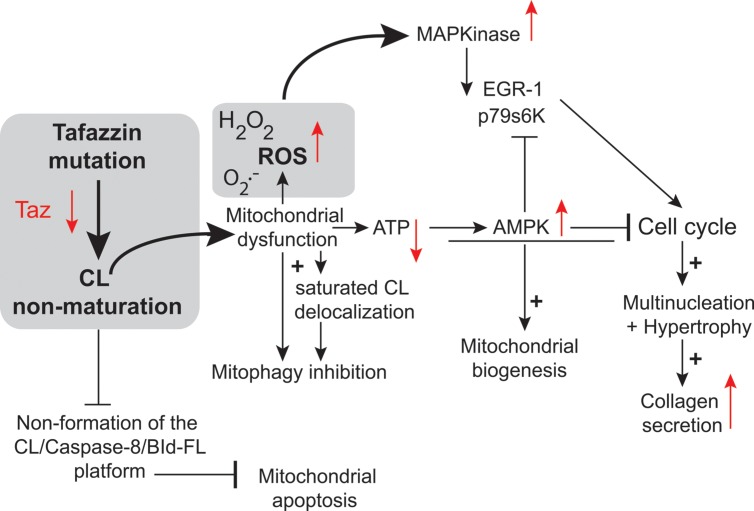
**Tafazzin knockdown in neonatal cardiac fibroblasts.** Tafazzin gene knockdown results in mitochondrial dysfunction, with the production of lower levels of ATP and higher levels of superoxide anions and associated ROS. ROS activate MAPKs, induce gene expression factors and cell cycle regulators and lead to cell cycle progression. Alternatively, ATP deficiency may activate AMPK, leading to mitochondrial biogenesis and the cessation of cell proliferation. Tafazzin knockdown ultimately results in multinucleation, hypertrophy and enhanced collagen secretion [this scheme is derived from the work of He and collaborators (He et al., 2013) and has been freely interpreted with permission of the American Journal of Physiology: Heart and Circulatory Physiology from the American Physiology Society, USA].

Two key studies relied on neonatal cardiac fibroblasts and cardiac myocytes (He, 2010; He et al., 2013). The authors found that *TAZ* knockdown resulted in the hypertrophy of neonatal cardiac myocytes (He, 2010). In this case, the authors used an adenovirus as the vector for transferring a tafazzin small hairpin RNA (shRNA) into neonatal ventricular myocytes (NVMs) for investigation of the effects of tafazzin knockdown. The tafazzin shRNA adenovirus consistently knocked down tafazzin mRNA and CL levels, and significantly decreased mitochondrial ATP production. The phosphorylation of AMP-activated protein kinase (AMPK) and the density of mitochondria were both higher in tafazzin-knockdown NVMs than in control cells into which a scrambled shRNA had been introduced. In assessments of hypertrophy (*in vitro* tafazzin knockdown), overall surface area, protein synthesis, and expression of the hypertrophic marker gene encoding brain natriuretic peptide were all higher in NVMs infected with the tafazzin shRNA adenovirus (He, 2010). According to Richter and Ruderman (2009) ATP depletion in cardiac myocytes has several detrimental effects, including contractile dysfunction, hypertrophy, and production of ROS potentially responsible for autophagy dysregulation and cell death. ATP depletion switches on ATP-producing catabolic pathways, such as fatty acid oxidation and glycolysis, and switches off ATP-consuming anabolic pathways, such as lipogenesis and glyconeogenesis. In the long term, mitochondrial ATP depletion may be compensated by an increase in mitochondrial “mass” due to enhanced mitochondrial biogenesis, mediated by activation of the “fuel gage” AMPK (**Figure 7**) (Hardie, 2008). Jager et al. (2007) reported that AMPK directly phosphorylated and activated PGC-1, the key regulator of mitochondrial biogenesis. Strength and endurance training leads to the consumption of larger amounts of ATP, associated with an increase in mitochondrial volume in the muscle (Reznick and Shulman, 2006). Tafazzin knockdown increases mitochondrial density in NVMs, consistent with the notion that ATP depletion enhances mitochondrial biogenesis (**Figure 2**).

This is also consistent with the large number of mitochondria found in the lymphocytes of patients with BTHS (Xu et al., 2005; Gonzalvez et al., 2013) (**Figure 2**). This greater mitochondrial density contributes to an increase in the volume of NVMs (cardiac myocytes have a very high mitochondrial density, likely to influence cell shape) (Goffart et al., 2004) as observed in *TAZ*-knockdown NVMs (thus, *TAZ* knockdown induced hypertrophy in cardiomyoctes).

In primary cultures of neonatal ventricular fibroblasts (NVFs) infected with a tafazzin short hairpin RNA adenovirus, tafazzin knockdown increased ROS production (He et al., 2013) and induced mitogen-activated protein kinases and protein and DNA synthesis via cell cycle regulators. It also reduced intracellular ATP levels, activated AMPK, and caused multinucleation, hypertrophy, and enhanced collagen secretion. Tafazzin knockdown interrupts the NVF cell cycle, potentially contributing to the fibrosis and dilated CM observed in BTHS. It is possible that knocking down tafazzin in different model systems would result in a different phenotype, because there appears to be no correlation between genotype and phenotype in BTHS. For instance, Tafazzin knockdown may have different effects at different stages of embryonic development.

## Use of Human Induced Pluripotent Stem Cells (Hipsc) for the Modeling of Cardiomyopathies

Advances in the understanding and treatment of cardiac disorders have been thwarted by the inability to study beating human cardiac cells *in vitro*, making it necessary to use other models, such as yeast, *Drosophila*, zebrafish, and animal or transfected cells (as described above). Furthermore, the invasive nature of the methods used to obtain human cardiac tissues has hindered research in this area, which has progressed much more slowly than hematological research, for example.

**FIGURE 8:**
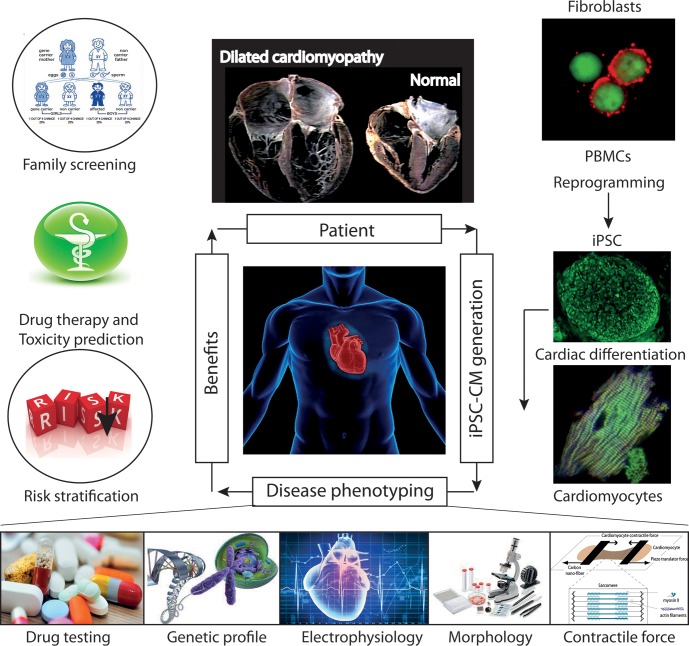
**Future role of iPSC-CMs in the clinical care of patients with hereditary cardiac disorders.** iPSC-CMs can be profiled by molecular techniques, and electrical and mechanical measurements, and on the basis of their response to various pharmacological agents. Such studies can provide information about the predicted severity of disease, associated risks, and response to therapy, which can be used to support clinical decision making. In addition, the functional characterization of the tissue might help to identify candidate causal variants and to facilitate the screening of family members for the disease. Abbreviations: iPSCs, induced pluripotent stem cells; iPSC-CMs, induced pluripotent stem cell-derived cardiomyocytes; PMBCs, peripheral blood mononuclear cells.

**FIGURE 9:**
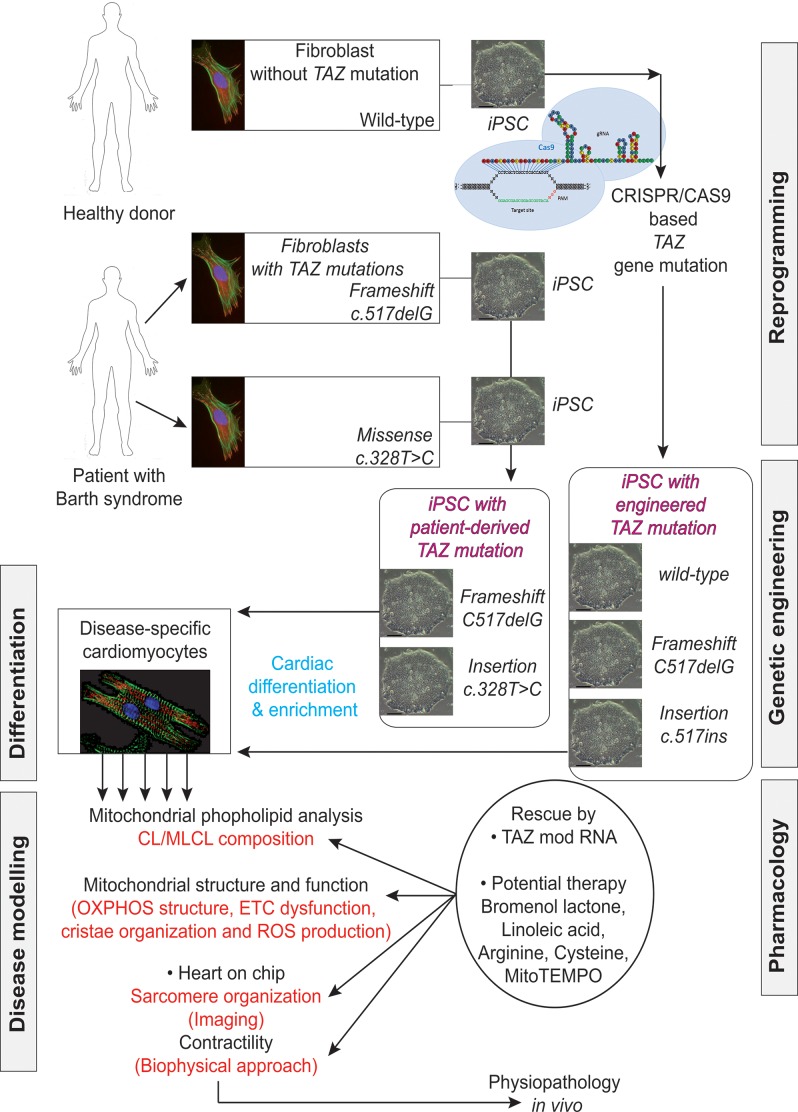
**Induced pluripotent stem cells generated from patients suffering from Barth syndrome.** Disease-specific mutations are introduced into wild-type iPSC lines, facilitating direct comparison with isogenic controls. iPSC-CMs are subjected to biochemical analyses, such as phospholipid analysis by mass spectrometry and mitochondrial structure/function analysis. iPSC-CMs are also used in “heart-on-chip” assays, to model the pathophysiology of the disease *in vitro* and to investigate the underlying mechanism of BTHS (particularly as concerns binding capacity or force characteristics during contraction) and to evaluate potential therapies involving untargeted or targeted antioxidants. [This figure was developed from a publication of Wang et al. (2014b) and from comments recently published concerning the missing link between mitochondrial ROS production and the destabilization of sarcomere organization, raising questions about the relationship between ROS and dilated LVNC in BTHS patients (Raval and Kamp, 2014; Zweigerdt et al., 2014)].

The recent development of iPSCs has now emerged as a reliable method for producing patient-specific somatic tissue cell lines via directed differentiation. iPSC-derived cardiomyocytes (iPSC-CMs) provide a robust platform for studying genetic cardiac disorders, because this approach makes it possible to analyze and manipulate human cardiac cells in culture (**Figure 8**). This model has already been shown to be complementary or superior to transfected cell or animal models of cardiac genetic disorders (**Figure 9**). iPSCs are produced by reprogramming somatic cells to yield a more pluripotent phenotype, with the capacity for self-renewal and differentiation into derivatives of all three germ layers: endoderm, mesoderm and epiderm [for reviews see (Matsa et al., 2014; Sallam et al., 2014)]. This behavior was first described by Takahashi and Yamanaka (2006) and then by Yu et al. (2007). Several groups subsequently showed that the directed differentiation of these cells could produce somatic cell derivatives, such as neurons, hematopoietic cells and cardiomyocytes (Dimos et al., 2008; Kambal et al., 2011; Mordwinkin et al., 2013).

Dey et al. (2009) and Slomka et al. (2012) recently performed a detailed characterization of multiple stem cells and showed that the differentiation of iPSCs led to a loss of immunogenicity, resulting in tolerance induction, despite differences in antigen expression between iPSC-derived cells and the original somatic cells (de Almeida et al., 2014). Human induced pluripotent stem cells (hiPSC) have been used to study the molecular mechanisms of long-Q-Tc syndrome (Moretti et al., 2010; Matsa et al., 2014), catecholaminergic polymorphic ventricular tachycardia (Itzhaki et al., 2012; Jung et al., 2012), dilated CM [for review; (Selem et al., 2013)], hypertrophic CM (Lan et al., 2013), LEOPARD syndrome (Lan et al., 2013), arrhythmogenic CM (Lombardi and Marian, 2010), Friedreich ataxia (Hick et al., 2013), hypoplastic left heart syndrome (Gaber et al., 2013; Kobayashi et al., 2014), Marfan syndrome (Quarto et al., 2012), other congenital heart diseases and, more recently, BTHS (Wang et al., 2014a).

Cardiovascular cells derived from patient-specific iPSCs harbor gene mutations associated with the pathogenesis of inherited cardiac diseases and congenital heart diseases. The differentiation of disease-relevant and patient-specific iPSCs into relevant cell types can relieve major limiting steps in disease modeling and investigation. This approach makes it possible to generate large amounts of appropriate cellular material for use in mechanistic studies. It is now possible to develop appropriate cell-based assays for assessing relevant aspects of the disease studied (**Figure 8**):

A recent publication combined analysis of human iPSCs cells together with genome editing, cell biology (including biochemical aspects and bioenergetic) and physiological approach to highlight new insights into BTHS-associated CM (Wang et al., 2014a).

Wang et al. (2014a) established patient-specific BTHS-iPSCs lines containing frameshift or missense mutations of the TAZ gene and used them to study BTHS. The authors used well-established protocols to direct the differentiation of these cell lines into cardiomyocytes. They tried to eliminate the effect of the differences in genetic background between the cells through three independent approaches:

- shRNA-mediated knockdown of *TAZ* in primary rat cardiomyocytes, to confirm the metabolic phenotype in primary cells from another species.
- Phenotypic rescue in BTHS hiPSC-CMs, by transfection with a synthetic modified RNA encoding wild-type tafazzin (Wang et al., 2014b).
- Cas9-mediated genome editing (Mali et al., 2013; Byrne et al., 2014) to introduce TAZ mutations into isogenic control hiPSC lines (**Figure 9**).

Wang et al. (2014a) then performed rigorous experiments on the patient-derived and genetically engineered iPSC cells by sarcomeric reconstitution and biophysical testing to assess the causal role of *TAZ* mutations in cardiac dysfunction associated with BTHS. This approach yielded highly impressive results including an *ex vivo* confirmation of structural and functional abnormalities linked to *TAZ* mutation, which ended in sparse and irregular sarcomeres with reduced mechanical capabilities.,

It is clear that by using patient-specific iPSC lines it is possible to obtain new mechanistic insights into the physiopathology of a mitochondrial CM associated with a single gene mutation and potential strategies for its treatment.

## Using iPSC Technology to Study Sarcomerogenesis

The differentiation of disease-relevant and patient-specific iPSCs into relevant cell types as described above now makes it possible to develop appropriate cell-based assays for assessing relevant aspects of the disease studied (**Figure 9**). Dudek et al. (2013) reported aberrant RC function in undifferentiated iPSCs obtained from three BTHS patients (derived from dermal fibroblasts) with different *TAZ* mutations - TAZ10, 590 G > T missense, severe BTHS; TAZ13, 110-1 AG > AC splice site, severe BTHS; TAZ15, 170G > T missense, mild BTHS. The diagnosis was based on analyses of the CL/MLCL ratio in the patients’ fibroblasts. BTHS-iPSCs reproduced the tafazzin deficiency on CL remodeling and mitochondrial defects in oxidative phosphorylation. These cells had a very low basal oxygen consumption rate but maximal respiratory capacity and this was accompanied by major changes in the structure of the RC supercomplexes, resulting in a massive increase in ROS production. Supercomplex disassembly, dysregulated respiration with aberrant ROS production (Gonzalvez et al., 2013) and respiration and mitochondrial deficiencies (Xu et al., 2005; Gonzalvez et al., 2013) have also been described in patient-derived BTHS lymphoblasts.

**FIGURE 10:**
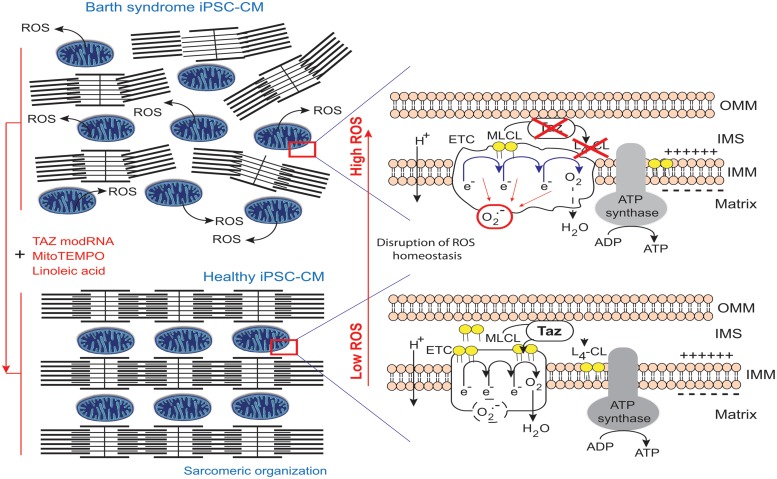
**Pathophysiology of Barth syndrome and iPSC-CMs.** Healthy iPSC-CMs have organized myofilaments and mitochondria with active transacylase tafazzin linked to the outer membrane and facing the intermembrane space (Brandner et al., 2005), with tafazzin in an intermembrane position (Herndon et al., 2013), allowing CL processing. This results in the conversion of monolysocardiolipin (MLCL; immature CL) to L4-CL (mature CL) in the IMM and the formation of normal electron transport chain (ETC) complexes and normal ATP synthase function. However, the ETC normally produces few superoxide anions in a process homeostatically controlled by endogenous mitochondrial antioxidant (e.g., MnSOD). By contrast, in BTHS, iPSC-CMs with mutant *TAZ* lack L4-CL. CL cannot be processed correctly and respiratory complexes do not form normally (as they have a lower compaction) resulting in ETC complex malfunction and abnormally high levels of ROS production (red circle), contributing to myofilament disarray and contractile dysfunction. By contrast, when the defect is complemented, the respiratory supercomplexes are at the right distance, compacted and superoxide ion production is low (dashed black circle) (Claypool et al., 2008). modRNA encoding tafazzin can rescue the disease phenotype, as can buffering mitochondrial ROS with mitoTEMPO or providing a precursor for L4-CL, linoleic acid (redrawn from Raval and Kamp, 2014).

In contrast to the above results, Wang et al. (2014a) reported that the mitochondria in BTHS-hiPSC-CMs were smaller and more fragmented displaying elevated basal oxygen consumption rate with an impaired electron transport. The discrepancy between the findings (Dudek et al., 2013) and those of Wang et al. (2014a) highlights the need to investigate the effects of disease-specific mutations carefully and precisely, not only in the particular cell type studied (presumed to show the disease phenotype), but also in undifferentiated cells or, even other differentiated derivatives, to delineate the tissue-specific effects, which may include phenotypic heterogeneity due to different mutations (**Figure 10**).

Mitochondria regulate cardiomyocyte differentiation (Hom et al., 2011), which involves the assembly of organized arrays of sarcomeres. BTHS-derived hiPSC-CM sarcomeres grown on an unpatterned gelatin substrate form much less regular assemblies than control hiPSC-CMs (**Figure 10**). The lack of a robust pipeline of therapeutic agents for treating heart disease is partly due to the lack of *in vitro* models reproducing the essential structure-function relationships of healthy and diseased myocardia. One up-to-date approach was to plate individual hiPSC-CMs onto micropatterned fibronectin rectangles designed to mimic the dimensions of human adult cardiomyocytes (Gerdes and Capasso, 1995) to develop a “heart-on-chip” (HOC) in which sarcomeres self-organize into a laminar myocardium. The resulting mimetic contractile muscular thin film (MTF) constructs are able to contract and curl (Grosberg et al., 2011, 2012). With this technique, cyclic stretching could be observed to activate gene expression profiles characteristic of pathological remodeling (with a decrease in α- to β-myosin heavy chain ratios for example) and induce maladaptive changes to myocyte shape and sarcomere alignment (McCain et al., 2013). The sarcomeres in the control iPSC-CMs initially extended serially along the entire length of the cell, whereas those in BTHS-iPSC-CMs were intermittent and sparse (**Figure 10**). In mechanical investigations based on this system, hiPSC-CMs from patient-derived cell lines had weaker contractile functions, while treatment with *TAZ* modRNA restored sarcomere regularity. Culture in glucose normalized ATP levels to an extent similar to that achieved with *TAZ* modRNA, but did not rescue sarcomere formation. These results are particularly interesting, given that lymphoblasts derived from BTHS patients display slightly higher levels of ATP production via glycolysis (i.e., in the presence of glucose) than control lymphoblasts, suggesting that the impairment of mitochondrial ATP production may be compensated temporarily by an increase in the size of the mitochondrial compartment (Gonzalvez et al., 2013). Combined with the sarcomere data, these later observations suggest that sarcomerogenesis is sensitive to mitochondrial function (especially during stem cells differentiation), regardless of whole-cell ATP levels (Chung et al., 2007).

It would be of interest to perform electrophysiological analyses to determine whether the observed *TAZ* mutation-dependent phenotypes result in impaired electromechanical coupling. Impaired coupling may underlie additional clinical signs in BTHS, such as ventricular arrhythmia and sudden cardiac death (Zweigerdt et al., 2014).

## Therapeutic Strategies

**FIGURE 11:**
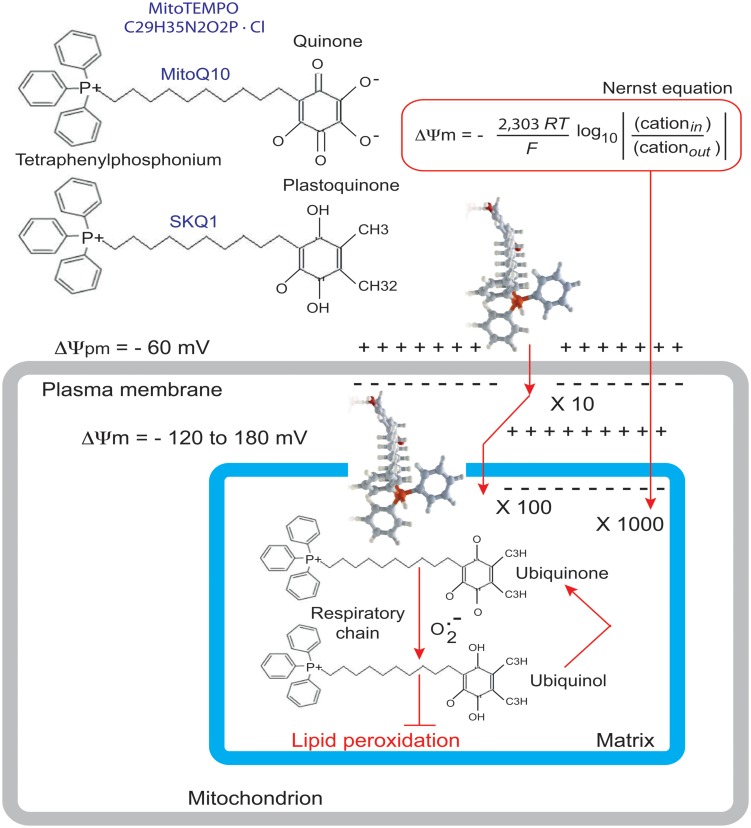
**Rationale for the accumulation of MitoQ10 or SKQ1 in cells and mitochondria.** The antioxidant component of MitoQ_10_ is the same ubiquinone found in coenzyme Q_10_ (Kelso et al., 2001), whereas the antioxidant of SKQ1 is a plastoquinone (Antonenko et al., 2008). Within mitochondria, the ubiquinone part of MitoQ is rapidly activated to yield the active ubiquinol antioxidant by the enzyme complex II (also called succinate dehydrogenase) in the mitochondrial respiratory chain (RC). As a result of detoxifying a free radical or other ROS, the ubiquinol part of MitoQ is converted to a ubiquinone, which is then recycled back to the active antioxidant by complex II within the mitochondria (James et al., 2005). It is this combination of a 1000-fold concentration within mitochondria, with conversion to the active antioxidant and recycling back to the active antioxidant after the detoxification of a free radical that makes MitoQ an effective mitochondrion-targeted antioxidant.

Wang et al. (2014a) used their BTHS-hiPSC-CMs disease model to test potential therapeutic strategies for BTHS. Valianpour et al. (2003) reported that fibroblasts from both BTHS patients and normal controls incubated with different concentrations of linoleic acid (a major CL precursor and an antioxidant) displayed dose- and time-dependent increases in CL levels. This was interpreted as evidence that increasing the amount of linoleic acid in the diet might be beneficial for patients with BTHS. Wang et al. (2014a) found that linoleic acid could rescue the defect in the organization and biophysical characteristics of sarcomeres. Moreover, using an antioxidant targeted to mitochondria, Mito TEMPO, they achieved a similar reorganization of the sarcomeres and rescue of function (**Figure 10**). Advances in mitochondrial research over the last decade have focused on the preservation of mitochondrial function, which is essential for cellular energy production, in the myocardium. Experimental and clinical trials have been conducted with various molecules targeted to the mitochondria, including MnSOD mimetics, such as EUK-8, EUK-134 and MitoSOD; choline esters of glutathione and *N*-acetyl-L-cysteine; triphenylphosphonium-ligated vitamin E, lipoic acid, plastoquinone and mitoCoQ_10_; and Szeto-Schiller (SS)-peptides (SS-02 and SS-31) (**Table 3**). Many of the results obtained have been inconclusive, but some, particularly those relating to mitoQ_10_ (very similar to the MitoTEMPO used by Wang’s team work) (Ajith and Jayakumar, 2014) have been informative in the sense that they point out the essential role of ROS. It may not be straightforward to translate these small-molecule interventions into patient treatment, even though related compounds are currently being tested in clinical trials [MitoQ for Parkinson disease, non-alcoholic fatty liver disease (NAFLD) and high levels of liver enzymes due to hepatitis C; SKQ1 for dry eye syndrome] (**Table 3**; **Figure 11**).

**Table 3:** Antioxidants targeted to mitochondria.

Antioxidant	Generic name
TEMPOL	4-hydroxy-2,2,6,6,tetramethylpiperidine-N-oxide
EUK-8, EUK-134	Salen derivative – inoganic MnSOD mimetics
Salen	Salen derivative – Mn(III) complex *o*-vanillin
Glutathione	Choline ester of glutathione
NAC	N-acetyl-1-cysteine
Vit. E	Vitamin E

Mitochondrially targeting with tetraphenylphosphonium (TPP^+^)	

MitoTEMPO (TPP^+^-1-hydroxy-3-carboxy-pyrrolidine)	
MitoQ10 (TPP^+^-Quinone10)	
Lipoic acid	
SKQ1, (TPP^+^-plastoquinone)	
MitoSOD, MnSOD	
Szeto-Schiller or SS-peptides (SS-02, SS-31)	


Taken together, these results provide an intriguing link between impaired sarcomere organization (and weak force generation) in cardiomyocytes and high levels of ROS production associated with the mitochondrial dysfunctions observed in cells with TAZ gene mutations. This is not particularly surprising, because ROS have been implicated in cardiomyocyte differentiation, sarcomerogenesis, and contractility (Chung et al., 2007; Hom et al., 2011; Steinberg, 2013).

## Perspectives

Despite major progress in our understanding of BTHS, there are still key unanswered questions to be addressed, over and above the potentially large differences between any two patient mutations studied. In particular, cells carrying similar mutations may display different patterns of contractile dysfunction in the “heart-on-chip” assay. Further clinical information is required concerning the features of the disease in the patients sampled. Moreover, current methods of iPSC generation mostly produce CMs with embryonic heart features, such as their dependence on glycolysis. It is unclear how relevant results obtained with embryonic-like CMs are to disease in adult hearts (**Figure 8**). Questions also remain as to whether the disease begins in and is limited to the mitochondria, or whether there are other functional impacts, on other compartments within cells, such as the ER or peroxisomes (Raja and Greenberg, 2014), which also contain CLs.

**FIGURE 12:**
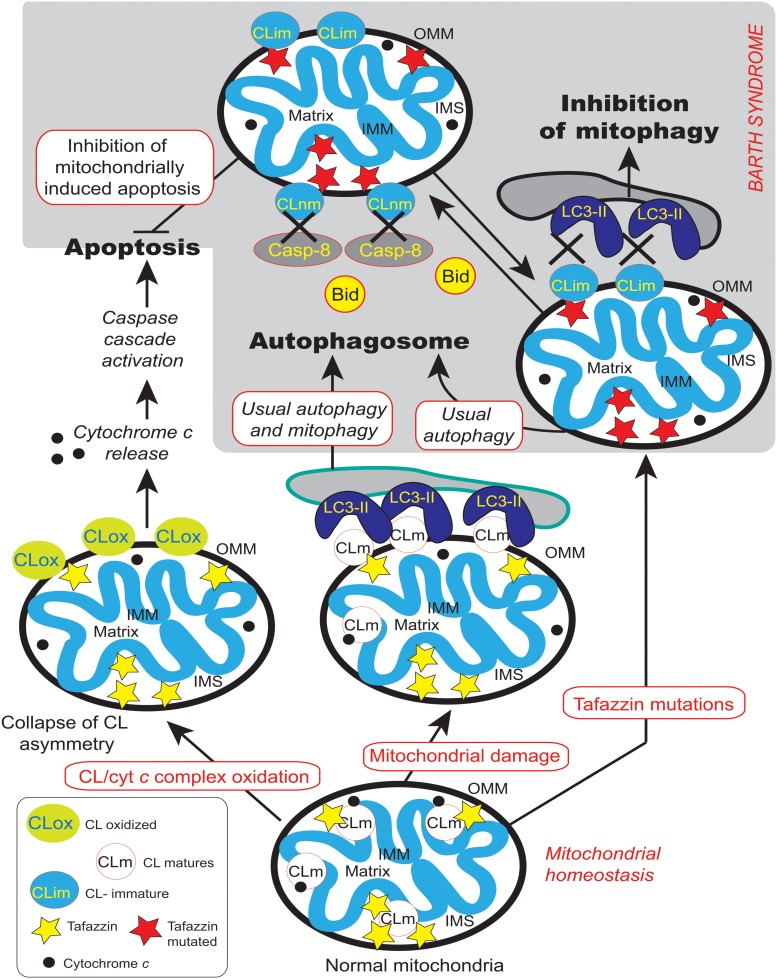
**Cardiolipin and the main transduction pathways for apoptosis and autophagy.** Collapse of cardiolipin content allows movements of CL from the inner mitochondrial membrane to the outer membrane required for both mitochondrially driven apoptosis and autophagic processes. If cardiolipin peroxidation is a prerequisite for the release of pro-apoptotic factors from the mitochondria into the cytosol, it could be problematic for mitophagy homeostasis. Moreover, changes in acyl chain composition associated with *TAZ* gene mutation and tafazzin dysfunction are directly involved in the inhibition of mitochondrially driven apoptosis (Gonzalvez et al., 2008, 2013) but has also be implicated in the possible inhibition of the mitophagic processes (Hsu et al., 2015). LC3-II is localized to preautophagosomes and autophagosomes, making this protein an autophagosomal marker (Kabeya et al., 2000) but also a marker of more specific mitophagosomes (that perform a specific type of autophagy called mitophagy). Normal and mutated tafazzin are represented by yellow and red stars, respectively, IMM, inner mitochondrial membrane; IMS, intermembrane space; OMM, outer mitochondrial membrane, LC3-II,microtubule-associated protein 1 light chain 3-II). A black cross marks the abolition of protein-to-protein interactions.

Tafazzin appears to be necessary for the execution of mitophagy (Hsu et al., 2015) and this observation may help to add some new directions to BTHS research, since an inhibition of mitophagy may lead to the accumulation of unwanted deleterious mitochondria in the patients’ tissues. Tafazzin is specifically required for the initiation of mitophagy whereas tafazzin depletion does not impair other autophagic processes. However, how does tafazzin regulate the initiation of mitophagy? One possible explanation was provided by Chu et al. (2013), who showed that externalization of CL from the IMM to the OMM surface serves as a recognition signal that directs damaged mitochondria to mitophagy (Chu et al., 2013, 2014). Indeed, the association between impaired mitochondrially driven apoptosis (Gonzalvez et al., 2008, 2013) and the inhibition of mitophagy (Hsu et al., 2015) due to TAZ mutation, CL non-maturation (**Figure 12**) and related ROS production is strictly linked to LVNC (**Figure 1**). Failure to maintain a critical balance between mitophagy and mitochondrial biogenesis or homeostatic turnover of mitochondria results in a population of dysfunctional mitochondria (and, in the case of BTHS, with immature CL and MLCL accumulation) that contribute to various disease processes. Inhibition of autophagy is emerging as a main actor together with apoptosis defects leading to LVNC.

Large mitochondrial DNA deletions, a common cause of mitochondrial diseases, have been detected in pluripotent stem cells (Van Haute et al., 2013) and during aging processes (Bratic and Larsson, 2013). An additional strategy is open to researchers, because the targeted engineering of the mitochondrial genome of iPSCs is now possible (Bacman et al., 2013). This makes it possible not only to model mitochondrial diseases in cell culture, but also to investigate the effects of the age-related or reprogramming-associated accumulation of heteroplasmic mutations in iPSCs. Such studies will provide answers to an important medical question, which is also highly relevant to BTHS: Are the iPSCs generated from older individuals of similar biological quality as those generated from younger cell sources, such as cord blood, or should the banking of primary cells be initiated at the neonatal stage, for the subsequent generation of clinically available, applicable products.

Mutations causing abnormalities of mitochondrial function and mtDNA are associated with common cardiac diseases, such as BTHS. Despite the promising mitochondria-targeted drugs, currently used at the cellular or tissue levels and emerging from the laboratory, very few such molecules have successfully completed clinical trials. Antioxidants ligated to tetraphenylphosphonium, such as vitamin E, lipoic acid, plastoquinone, MnSOD, and mitochondrial CoQ_10_, have been experimentally shown to be effective for attenuating the mitochondrial oxidative stress associated with such diseases. MitoQ_10_, which targets mitochondria, could provide a novel approach to decreasing oxidative damage and has the potential to become a new therapeutic agent for use in humans. These strategies suggest that the pharmacological restoration of a normal or upregulated mitophagy could provide a novel additive treatment of the pathology. However, currently there is too little evidence from randomized trials to advance a recommendation for systematic use of antioxidant supplementation to prevent heart disease. As mitochondrial damage is a key element of the pathophysiology of heart disease in general, various approaches based on the targeting of antioxidant compounds to mitochondria should be explored, for the development of new treatments for cardiomyopathies linked to BTHS.

## Conclusion

There has been a substantial improvement of our understanding of the molecular mechanisms underlying the BTHS pathology and there is an increasing hope for future efficient therapeutic treatments. However, many additional studies will be required before mitochondria-targeted treatments can be made available for the prevention and treatment of diverse cardiomyopathies. Such basic work will help us understand the disease mechanism and its numerous phenotypes.

## Conflict of Interest Statement

The authors declare that the research was conducted in the absence of any commercial or financial relationships that could be construed as a potential conflict of interest.
